# Unraveling Hierarchical Structure in Ionic Liquid–Extractant Mixtures with X‑ray Scattering

**DOI:** 10.1021/acs.jpcb.6c04769

**Published:** 2026-10-26

**Authors:** Xiaoyu Wang, Allison A. Peroutka, Soenke Seifert, Michael J. Servis

**Affiliations:** † Ralph E. Martin Department of Chemical Engineering, 3341 Univeristy of Arkansas, Fayetteville, Arkansas 72701, United States; ‡ Chemical Sciences and Engineering Division, 1291 Argonne National Laboratory, Lemont, Illinois 60439, United States; § X-ray Science Division, Argonne National Laboratory, Lemont, Illinois 60439, United States

## Abstract

Liquid–liquid extraction (LLE) is the predominant method for the selective recovery and separation of many critical materials, including rare earth elements and platinum group metals. Ionic liquids (ILs) have been proposed to replace traditional solvents in LLE to enhance or manipulate separation performance. The tunable nanostructure of ILs is a unique and defining characteristic that impacts their function, but how that structure is affected by mixing with the LLE extractant is not understood. To investigate this, we combine small-angle X-ray scattering (SAXS) and molecular dynamics (MD) simulations. SAXS profiles computed from the MD simulations are in good agreement with the experimental data. Known pure IL nanostructures persist upon the addition of the malonamide extractant. Decomposition of the simulation structure factors shows extractant distribution between the domains of the IL solvent. In addition, these mixtures also show strong composition fluctuations at low *q*, indicating inhomogeneity related to phase instability between IL and the extractant. In a trend exactly opposite to that observed in conventional molecular alkane solvents, increasing the alkyl tail length in ILs depresses the fluctuations, suggesting that enhanced IL hydrophobicity promotes mixture stability. These findings suggest that traditional ligand design strategy is, in fact, counterproductive and needs to be reevaluated for LLE processes using ILs. This study provides the basis for understanding the fundamentals of IL–extractant mixtures and will lead to the rational design of both IL and the extractant for more efficient separation processes.

## Graphical abstract

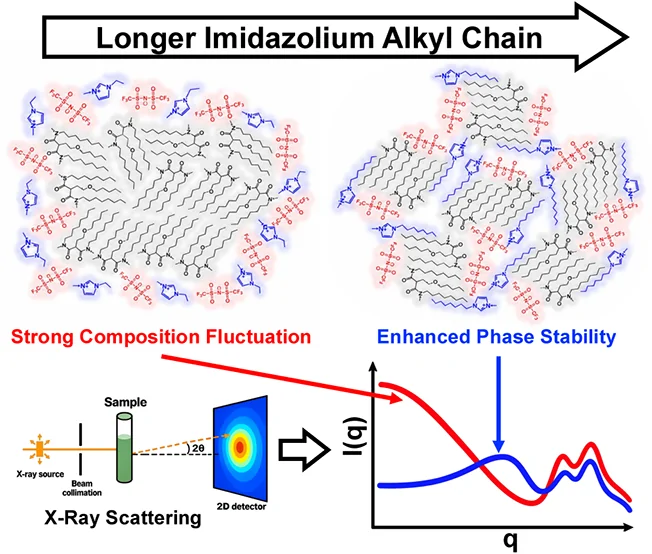

## 1 Introduction

Liquid–liquid extraction (LLE) is the predominant industrial method for the selective recovery and separation of critical metals, including rare earth elements (REE) and platinum group metals, which have wide applications in electronic devices, laser instruments, and magnetic materials.^1–4^ In traditional LLE of metal ions, these ions are selectively transferred from an aqueous solution to an immiscible organic phase by an extractant molecule. The amphiphilic extractant molecule is designed to bind to the rare earth metal ion through the polar headgroup, whereas the apolar tail group maximizes hydrophobicity to make the metal-extractant complex soluble in the organic phase to achieve separation.^5,6^ One limitation of LLE is third phase formation, where the organic phase can undergo liquid–liquid phase splitting after sufficient metal loading, with one phase containing most of the REE-extractant species and the other phase mainly consisting of the solvent.^7,8^ The formation of the dense organic phase limits extraction capacity, as it is incompatible with industrial process equipment. This phase transition has been connected to aggregation in the organic phase.^9–22^

Ionic liquids (ILs) are an alternative class of solvents that have been investigated for their use as the immiscible organic phase in LLE of metal ions.^23,24^ Independent of LLE applications, ILs are well-known for their novel liquid properties, including nanostructures that can be tailored through IL molecular design.^25^ How the IL nanostructure impacts LLE applications is not well-studied. Understanding how extractant molecules assemble, aggregate, and interact with the nanodomain structure inherent to the IL is vital to designing both ILs and extractants for better LLE processes.

X-ray scattering, including small-angle X-ray scattering (SAXS) and wide-angle X-ray scattering (WAXS), is a powerful technique for probing the microscopic liquid structure that has been applied to ILs.^25,26^ WAXS captures short-range structural features, including nearest-neighbor correlations, while SAXS is sensitive to long-range nanoscale organization and heterogeneity. These structural measurements are often paired with molecular dynamics (MD) simulations to provide a molecular interpretation. While simulations can be used to predict the total scattering intensity, *I*(*q*), they can also provide partial structure factors, *S*(*q*), to decompose the total scattering that is analogous to what is observed experimentally.

In a typical analysis, when the simulated total scattering profile agrees with the experimental one, the MD trajectory is then used retrospectively to identify the molecular origins of specific scattering features. For ILs, this combined approach is especially valuable because it can interpret experimental scattering data and provide information on its unique liquid structure across different characteristic length scales. At short length scales, mid-to-high-*q* scattering features in WAXS reflect local ionic adjacency and charge alternation between cations and anions.^27,28^ At intermediate length scales, many imidazolium-based ILs exhibit a SAXS prepeak associated with polar/apolar nanodomain segregation, which arises from the coexistence of charged ionic groups and nonpolar alkyl chains.^29–31^ By decomposing the simulated *S*(*q*), MD simulations can identify which atomic or molecular groups contribute to these features and how changes in molecular design, such as cation alkyl-chain length or extractant tail structure, alter the IL nanostructure.

At larger length scales, the low-*q* SAXS patterns can include contributions from concentration fluctuations for liquid mixtures near a liquid–liquid phase instability. This has been observed for various IL/organic mixtures.^32–34^ These fluctuations dominate the low-*q* scattering response and can be quantitatively characterized by the correlation length, *ξ*. According to the Ornstein–Zernike (OZ) scattering model, the low-*q* behavior in the SAXS can be described by eq 1.^35–37^

$$I\left(q\right)=\frac{I_{\mathrm{oz}}}{1+{\left(q\xi_{\mathrm{oz}}\right)}^{2}}+B$$ 1

where *I*_oz_ and *ξ*_oz_ are the intensity and correlation length, respectively, of the fluctuations; B is the constant background scattering. Thus, the fitted *I*_oz_ and *ξ*_oz_ provide quantitative measurements of concentration fluctuations, with larger values indicating stronger spatial heterogeneity.

In this work, we combine synchrotron-based SAXS measurements and MD simulations to understand how extractant molecules interact with IL domain structures. We study a system containing model imidazolium trifluoromethanesulfonimide based ILs and a standard malonamide extractant for REEs separation, N,N′-dimethyl-N,N′-dioctylhexylethoxy malonamide (DOHE). Molecular structures and nomenclatures of all ILs and DOHE covered in this work are drawn in Figure 1. Good agreement is obtained between the SAXS profiles calculated from MD simulations and the experimental measurements, particularly in the low-*q* region, providing confidence in using the MD trajectories to interpret the molecular origins of the scattering features. In contrast to prior work,^38^ we establish that the long-range structure present in these IL/extractant mixtures is governed by composition fluctuations related to the phase instability between those two components. OZ analysis of the low-*q* SAXS profiles shows that increasing the IL cation alkyl chain length reduces the concentration fluctuations in the IL-DOHE mixture, suggesting a more apolar character in the IL enhances the stability of the mixture. Furthermore, *S*(*q*) breakdown from MD shows that the IL-DOHE prepeak grows with increasing IL cation alkyl-chain length, indicating that DOHE associates more strongly with the more prominent IL apolar domains. These results suggest how extractant and IL structure influence the nanostructure of their mixtures and govern their phase stability, providing useful design principles for IL-based separation systems.

**Figure 1:**
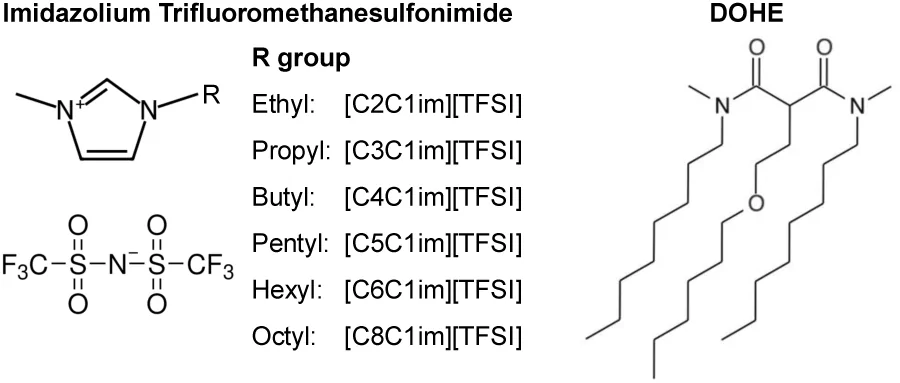
Molecular structures and nomenclatures of ILs and extractant investigated in this work.

## 2 Experimental and Computational Methods

### 2.1 Materials and Sample Preparation

All ILs were purchased from Iolitec (99.5%) and DOHE was purchased from Technocomm Ltd. (99%), both used as received. Samples were prepared by mass, assuming no excess volume upon mixing. One mL samples were hand vortexed and centrifuged to ensure dissolution. These were then loaded into 2.0 mm outer-diameter quartz capillaries for scattering measurements.

### 2.2 X-ray Scattering Measurements

SAXS/WAXS data from all compositions were taken at beamline 12-ID-E at the Advanced Photon Source (APS) using an 18 keV X-ray energy. Scattering patterns for both SAXS and WAXS regions were measured simultaneously, averaging over five 0.1 s exposures. Using a temperature controlled cell, SAXS/WAXS measurements were taken at 298 K (25 °C) or at various temperature steps from 298 K (25 °C) to 353 K (80 °C). After empty capillary subtraction, SAXS patterns were normalized to an absolute scale using the SAXS intensity of pure water at 25 °C. WAXS patterns were then normalized based on their overlap with the corresponding SAXS patterns.

### 2.3 Molecular Dynamics Simulations

The General AMBER Force Field (GAFF2)^39,40^ is used to describe intra- and intermolecular interactions for DOHE and ILs. Atomic partial charges for these molecules are first derived using the RESP method^41^ from DFT calculations at the B3LYP^42,43^ level of theory with the 6-31G­(d,p) basis set in GAUSSIAN 16.^44^ The partial charges of each IL ion pair are then scaled by 0.9 to account for polarizability.^45,46^ Geometric mixing rules are used for all cross-term Lennard-Jones interactions. An energy cutoff of 12 Å is used for nonbonded Lennard-Jones and electrostatic interactions. Long-range electrostatic interactions are accounted for using a particle-particle particle-mesh solver.^47^ The leapfrog stochastic dynamics integrator is used at 350 K with a time step of 2 fs.^48^ The Parrinello-Rahman barostat^49,50^ is applied at 1 atm. All simulations are performed for 2 μs, with the first half used for equilibration and the remaining 1 μs used for *I*(*q*) and *S*(*q*) sampling. The MD calculations, as well as *I*(*q*) and *S*(*q*) analysis, are carried out using the GROMACS software package.^51^

### 2.4 Calculations of *S­(*q*)* and *I­(*q*)* from Simulations

The total X-ray scattering intensity *I*(*q*) can be calculated from MD simulations by eq 2.

$$\begin{array}{ll} I\left(q\right) & =\frac{1}{N}\{\left[\overset{N}{\underset{i=1}{\sum }}f_{i}\left(q\right)\cos\left(q\cdot r_{i}\right)\right]\cdot \left[\overset{N}{\underset{j=1}{\sum }}f_{j}\left(q\right)\cos\left(q\cdot r_{j}\right)\right]\\ & +\left[\overset{N}{\underset{i=1}{\sum }}f_{i}\left(q\right)\sin\left(q\cdot r_{i}\right)\right]\cdot \left[\overset{N}{\underset{j=1}{\sum }}f_{j}\left(q\right)\sin(q\cdot r_{j})\right]\} \end{array}$$ 2

where *N* is the total number of atoms in the simulation box, ***r***_i_, ***r***_j_ are the Cartesian coordinates of atom *i*, *j*, and *f*_*i*_(*q*), *f*_*j*_(*q*) are the form factors of atom *i*, *j* at *q*. The simulated *I*(*q*) profiles are placed on the same water referenced intensity scale for comparison with experiment. Because the experimental and simulated intensities are normalized through different procedures, systematic differences in absolute intensity may remain.

In addition to the experimentally measurable *I*(*q*), the structure factor (*S*(*q*)), calculated through eq 3, is often used to identify and characterize short- and long-range structural features of ILs.

$$S\left(q\right)=\frac{1}{N}\left\{\left[\overset{N}{\underset{i=1}{\sum }}\cos\left(q\cdot r_{i}\right)\right]\cdot \left[\overset{N}{\underset{j=1}{\sum }}\cos\left(q\cdot r_{j}\right)\right]+\left[\overset{N}{\underset{i=1}{\sum }}\sin\left(q\cdot r_{i}\right)\right]\cdot \left[\overset{N}{\underset{j=1}{\sum }}\sin\left(q\cdot r_{j}\right)\right]\right\}$$ 3

In MD simulations, *S*(*q*) can be decomposed into individual groups of atoms, providing information on how these subsets contribute to the overall *S*(*q*). For example, for two subsets α and β in the simulation, the total structure factor of α + β (*S*_(*α+β*)(*α+β*)_(*q*)) can be decomposed into self-contributions (*S*_*αα*_(*q*), *S*_*ββ*_(*q*)) and cross-contributions (*S*_*αβ*_(*q*), *S*_*βα*_(*q*)), which are denoted in eq 4

$$S_{\left(\alpha +\beta )(\alpha +\beta \right)}\left(q\right)=S_{\alpha \alpha }\left(q\right)+S_{\beta \beta }\left(q\right)+S_{\alpha \beta }\left(q\right)+S_{\beta \alpha }\left(q\right)$$ 4

In addition, since *S*_(*α+β*)(*α+β*)_(*q*) is symmetric with respect to its components, the two mutual cross contributions should be identical to each other:

$$S_{\alpha \beta }\left(q\right)=S_{\beta \alpha }\left(q\right)$$ 5

## 3 Results and Discussion

### 3.1 Simulated and Experimental SAXS Profiles of Pure ILs

To evaluate how well the MD simulations agree with the experimental SAXS profiles in these IL solvent systems, we first investigate pure ILs before moving on to binary mixtures. Figure 2 shows the simulated SAXS profiles obtained using the GAFF force field with 0.9-scaled partial charges for the IL ion pairs, which reproduce the characteristic structural features observed experimentally for pure imidazolium-based ILs. Our simulations also capture the change between the different imidazolium cations: A prepeak near *q* = 0.3 Å^–1^ in the low-*q* region becomes more pronounced as the alkyl chain length on the imidazolium cation increases. The microscopic origin of this prepeak has been previously investigated by Margulis et al., who showed that, through *S*(*q*) decomposition, the growth of the prepeak with increasing alkyl chain length is mainly associated with the increasing length scale of nanoscale segregation between polar and apolar domains in ILs.^27,29^ Therefore, we do not repeat the decomposition here but use this established interpretation to support our assignment of the observed SAXS features.

**Figure 2:**
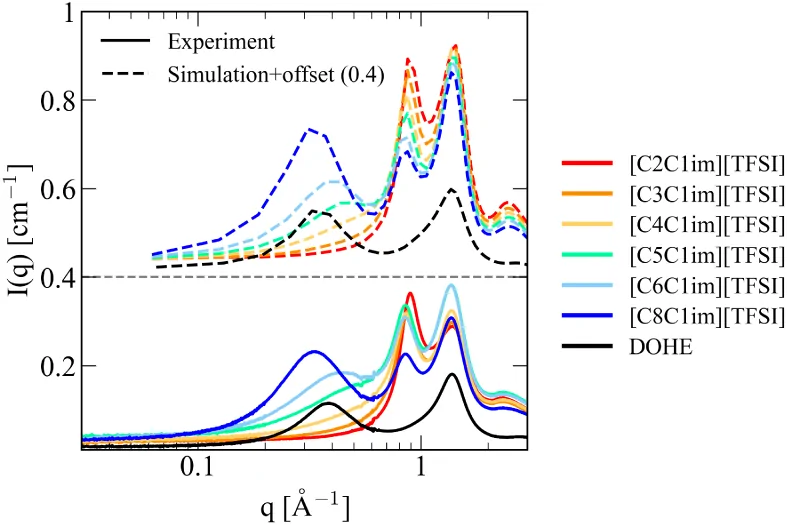
Experimental (solid lines, 25 °C) and simulated (dashed lines, 80 °C) SAXS profiles of pure imidazolium [TFSI] ionic liquids with varying cation alkyl-chain lengths and pure DOHE. The profiles are overlaid on a semilogarithmic scale with a logarithmic *q*-axis and a linear *I*(*q*)-axis. The simulated profiles are vertically offset by 0.4 cm^–1^ for clarity.

Representative simulation snapshots, shown in Figure 3, illustrate the development of the IL nanostructure with increasing alkyl chain length. As the alkyl tail becomes longer, the apolar regions become more pronounced and spatially extended (shown in gray), while the charged imidazolium and anion groups (shown in blue and red) remain organized within the polar network. In summary, the SAXS comparison and simulation snapshots confirm that the MD simulations capture the key nanoscale structural features of the pure ILs. This validation also supports the use of simulated SAXS to analyze component decomposition and domain formation in DOHE-IL mixtures.

**Figure 3:**
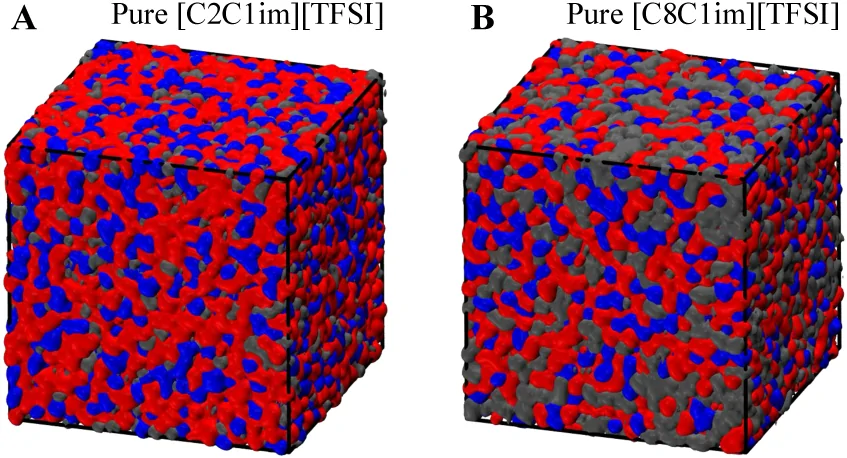
Snapshots of pure [C2C1im]­[TFSI] (A) and [C8C1im]­[TFSI] (B). Blue: cation headgroup. Gray: alkyl chain on cation. Red: anion.

### 3.2 Composition-Dependent Fluctuations

To understand how DOHE disrupts the liquid structure and domain architecture of the IL, we begin by examining the structural properties of binary DOHE/[C2C1im]­[TFSI] mixtures. The alkyl tail of this IL cation is sufficiently small that no meaningful apolar domain formation occurs (see Figure 2). Therefore, introducing DOHE, an amphiphilic molecule exhibiting strong polar and apolar interactions, is expected to substantially perturb the liquid structure and domain organization in IL, particularly through its apolar interactions. As shown in Figure 4A, we observe that the total scattering is dominated by the monotonically increasing intensity when *q* → 0. Additionally, this feature in the low-*q* region is significantly influenced by the DOHE concentration in the binary DOHE/IL mixture. We propose that this structure is the composition fluctuations that form in binary extractant/solvent mixtures in conventional LLE systems with molecular solvents^9,13,52,53^ or mixtures of IL and molecular solvents near a phase instability.^32–34^ In this low-*q* region, the scattering from composition fluctuations is governed by the curvature of the mixing free energy, reflecting the system’s proximity to a liquid–liquid demixing instability; at the spinodal, these fluctuations diverge.

**Figure 4:**
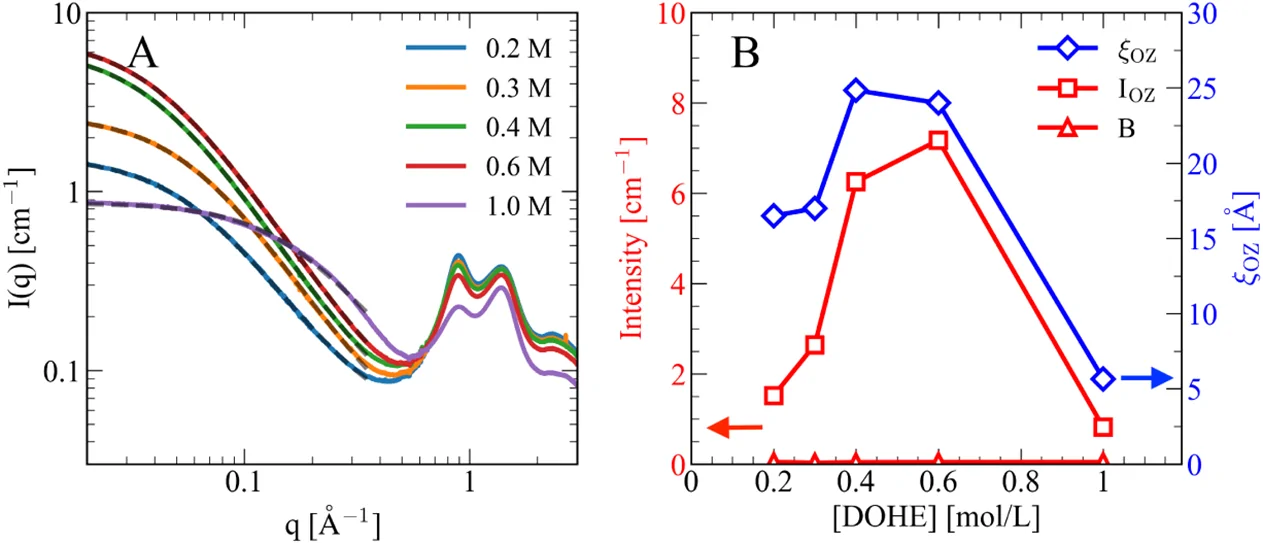
(A) Experimental SAXS/WAXS scattering profiles of DOHE in [C2C1im]­[TFSI] at 25 °C for DOHE concentrations between 0.2 and 1.0 M. Dashed lines show fits of the low-*q* region (0.02 ≤ *q* ≤ 0.35 Å^–1^) to the OZ form (eq 1). (B) Concentration dependence of the fitted OZ parameters, the fluctuation correlation length *ξ*_oz_, zero-angle scattering intensity *I*_oz_, and background scattering *B*.

To quantify the composition fluctuations, we fit the low-*q* region of the scattering pattern for each [DOHE] in [C2C1im]­[TFSI] with eq 1 in Figure 2, and plot the fitted parameters in Figure 4B. The fluctuation intensity and correlation length pass through a maximum at intermediate [DOHE] before decaying at high and low [DOHE]. This behavior is consistent with our previous studies on molecular diluents,^9,13,52,53^ which showed a maximum in fluctuations near 25% DOHE by volume. The location of this maximum is qualitatively consistent with the critical composition by volume through the Flory–Huggins model $\left(\phi_{\mathrm{DOHE}}^{c}=1/\left[1+\sqrt{V_{\mathrm{DOHE}}/V_{\mathrm{IL}}}\right]\right)$ in that the critical composition shifts toward a lower volume fraction of the component with the larger molecular volume^9^ (*V*_DOHE_ = 876.5 Å^3^/ molecule;^54^ [C2C1im]­[TFSI] *V*_IL_ = 426.7 Å^3^/molecule). Altogether, the similarity to prior work with molecular solvents suggests that varying extractant concentration induces a nonmonotonic trend in the correlation length as the solution transitions from IL-rich to DOHE-rich compositions.

### 3.3 Scaling Laws and Temperature-Dependent SAXS

Composition fluctuations have a unique temperature response that allows for their clear identification. Specifically, those fluctuations scale with temperature in a distinct manner, where the parameters obtained from the OZ fits follow power laws given by

6 $$\xi_{\mathrm{oz}}=\xi_{0}{|\frac{T-T_{\mathrm{sp}}}{T_{\mathrm{sp}}}|}^{-\nu }$$

and

7 $$I_{\mathrm{oz}}=I_{0}{|\frac{T-T_{\mathrm{sp}}}{T_{\mathrm{sp}}}|}^{-\gamma }$$

Here, *ξ*_oz_ and *I*_oz_ are fitted at a given temperature *T* using eq 1, while the prefactors *ξ*_0_ and *I*_0_ are system-specific constants. The scaling describes how the scattering intensity and correlation length vary with the reduced temperature (the distance from the spinodal temperature *T*_sp_), as defined by the exponents ν and γ, respectively.

In Figure 5A, we fit the temperature-dependent scattering and plot the fitted parameters from each temperature in panels Figure 5B–C. From the present data, we are not able to determine if the scaling exponents are more mean field-like (ν = 0.5, γ = 1) or 3D Ising-like (ν ≈ 0.63, γ ≈ 1.24); fitting both values of the exponents during the fit yields similarly reasonable results. As the sample is heated from room temperature (25 °C) to 80 °C, both *ξ*_oz_ and *I*_oz_ show systematic temperature dependence that is well described by the corresponding scaling forms over the measured range. The decrease in the fluctuation intensity with increasing temperature indicates a UCST-type phase diagram, where the liquid demixes upon cooling. Plotting *ξ*_oz_ and *I*_oz_ as functions of temperature, we fit them simultaneously to eq 6 and eq 7, respectively, using one shared *T*_sp_ for each scaling model. Simultaneous fits yield *ξ*_0_ = 8.5 ± 0.1 Å, *I*_0_ = 0.75 ± 0.02 cm^–1^, and *T*_sp_ = −20.1 ± 0.9 °C for the 3D Ising model. For the mean-field model, the corresponding values are *ξ*_0_ = 8.8 ± 0.1 Å, *I*_0_ = 0.78 ± 0.02 cm^–1^, and *T*_sp_ = −8.1 ± 0.9 °C. The uncertainties represent 95% confidence intervals. Fit residuals are provided in the Supporting Information. As this 0.4 M DOHE composition is somewhat near the critical composition (see Figure 4), these temperature estimates are likely close to the critical temperature. Because the fitted *T*_sp_ values lie outside the experimental range, they should be interpreted as model dependent extrapolations rather than direct measurements of the critical temperature.

**Figure 5:**
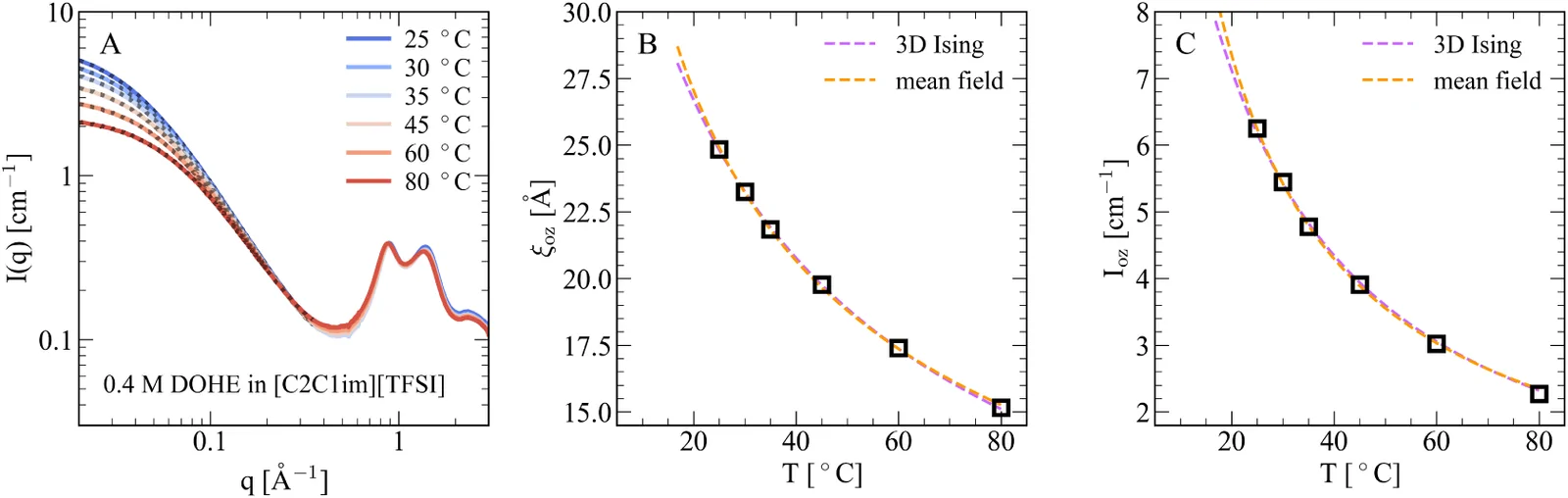
(A) Experimental SAXS/WAXS scattering profiles for 0.4 M DOHE in [C2C1im]­[TFSI] measured between 25 and 80 °C. Dashed lines show fits of the low-*q* region (0.02 ≤ *q* ≤ 0.35 Å^–1^) to the OZ form (eq 1), yielding the correlation length *ξ*_oz_ and zero-angle scattering intensity *I*_oz_. Temperature dependence of *ξ*_oz_ (B) and *I*_oz_ (C), together with power-law fits using fixed critical exponents for the 3D Ising universality class (ν = 0.63, γ = 1.24) and mean-field theory (ν = 0.50, γ = 1.00).

The temperature-scaling experiments for different IL tail lengths show that the fluctuation structural feature applies across the temperature range considered, but the magnitude of the fluctuations is modulated by how much the temperature variation brings the sample closer to or farther from the spinodal temperature. Only the [C2C1im]­[TFSI] mixture has a close enough spinodal temperature to observe a strong variation in the composition fluctuations over the 25 °C to 80 °C range used. However, in the Figure 6, we show temperature-dependent scattering for [C4C1im]­[TFSI], [C6C1im]­[TFSI], and [C8C1im]­[TFSI], with presumably lower *T*_sp_, such that the fluctuation scaling is negligible over the 25 °C to 80 °C range. This observation further suggests that longer-tailed IL cations more effectively accommodate amphiphilic DOHE extractant molecules, leading to suppressed composition fluctuations and improved phase stability. These data also show that the temperature primarily affects the amplitude of the low-*q* fluctuations, while the apolar domain, charge alternation, and adjacency features remain comparatively insensitive to temperature over this range. In the following, we proceed with MD simulations at elevated temperatures to improve sampling, with the understanding that the fundamental structural origins translate to room temperature experiments. Elevated-temperature simulations are desirable because IL systems often exhibit slow diffusion at low temperatures; increasing the temperature can therefore improve sampling within feasible simulation times. This provides confidence that the *S*(*q*) decomposition obtained from elevated-temperature MD simulations can be used to gain insight into the structural organization and component specific interactions between the IL and DOHE.

**Figure 6:**
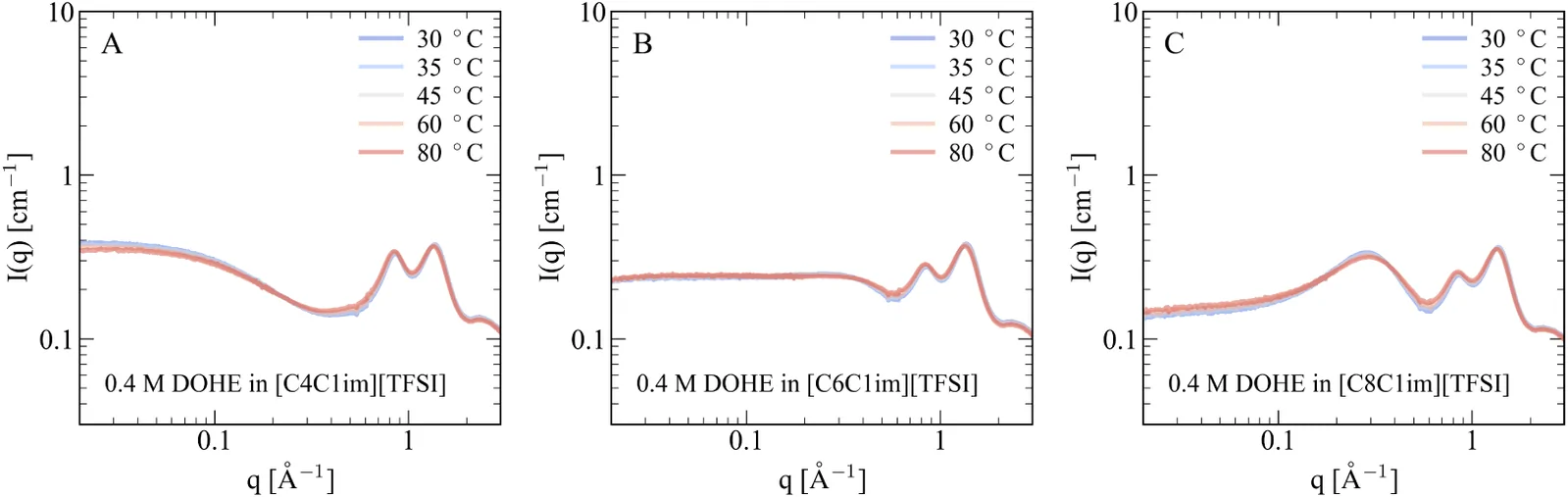
Experimental SAXS/WAXS scattering profiles for 0.4 M DOHE in [C4C1im]­[TFSI] (A), [C6C1im]­[TFSI] (B) and [C8C1im]­[TFSI] (C), measured between 25 and 80 °C.

To assess the possibility of other forms of mesoscale aggregation and strengthen the fluctuation interpretation, we also consider Guinier fits for representative 0.4 M DOHE mixtures for IL cation tail lengths resulting in mixtures both near and far from demixing (see Supporting Information). For the 0.4 M DOHE/[C2C1im]­[TFSI] mixture, the Guinier and OZ fits are nearly indistinguishable because the characteristic length is smaller and both models have similar leading behavior in the low-*q* limit. For the 0.4 M DOHE/[C2C1im]­[TFSI] system near the phase instability, as evidenced by its temperature scaling behavior in Figure 5, the Guinier model fails to reproduce the low-*q* curvature, whereas the OZ model describes the data well. As the characteristic length increases in the DOHE/[C2C1im]­[TFSI] mixture, the difference between the two forms becomes apparent. This suggests far from the phase instability, other scattering models could provide similarly good fits as the OZ form. Taken in isolation, such a comparably good fit might suggest ambiguity in the structural interpretation. However, the clear trends provided above in temperature, DOHE concentration, and IL cation tail length, when taken together, suggest all of the SAXS data support the simple explanation for low-*q* structure in DOHE/IL mixtures resulting from composition fluctuations. While some combination of OZ and another scattering contribution far from demixing cannot be disproven, we find no additional low-*q* structural mechanism is generally needed to rationalize our results.

### 3.4 Validation of Simulated SAXS and Cation-Dependent Domain Structure

The DOHE/IL mixtures are characterized by several structural patterns that contribute additively to the total SAXS patterns of the liquid. In the preceding sections, we revisited known nanostructural features of pure IL and showed how DOHE/[C2C1im]­[TFSI] mixtures feature additional low-*q* structure related to phase instability. Now, we systematically increase the cation alkyl tail size in both experimental and simulation scattering to observe its impact on all scattering peaks resulting from, in order of increasing *q*, the composition fluctuations at *q* → 0, the low-*q* domain ordering, and charge alternation in the WAXS region. The experimental and simulation SAXS patterns for 0.4 M DOHE in IL with cation tail lengths up to octyl ([C8C1im]­[TFSI]) are plotted in Figure 7. Overall, the simulations reproduce the principal peak positions and overall shapes of the experimental profiles, allowing the MD trajectories to be used to analyze the molecular origins of the observed structural features.

**Figure 7:**
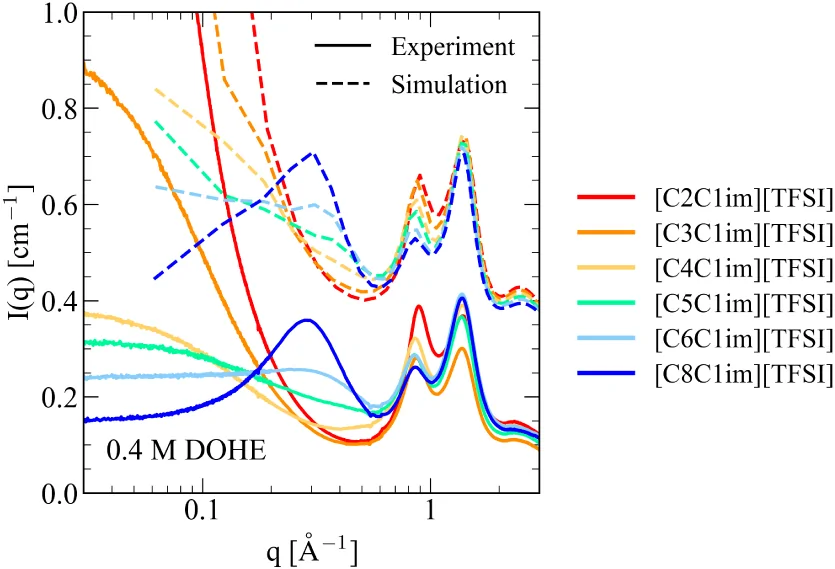
Experimental (solid lines) and simulated (dashed lines) SAXS profiles of 0.4 M DOHE in imidazolium TFSI ILs with varying cation alkyl chain lengths. The experimental measurements were collected at 25 °C, while the simulations were performed at 80 °C. The simulated profiles are offset by 0.3 for clarity.

The cation tail length impacts all three structural features. First, the systematic suppression of the low-*q* scattering intensity with increasing imidazolium cation tail lengths suggests that more apolar IL cations enhance mixture compatibility and improve phase stability. Second, the peak near *q* = 0.3 Å^–1^ becomes more pronounced as the alkyl-chain length increases, consistent with the prepeak observed in pure ILs. This behavior is attributed to the enhanced apolar–apolar domain structure formed by the longer alkyl chains. A similar trend has also been reported by Kashyap et al.^27^ for pure IL systems.^27^ Lastly, the charge alternation peak near *q* = 0.8 Å^–1^ decreases with increasing apolar tail length. These structural features are visualized in Figure 8, which compares 0.4 M DOHE in [C2C1im]­[TFSI] and [C8C1im]­[TFSI]. The snapshots show the increased segregation of DOHE and IL for [C2C1im]­[TFSI] compared to [C8C1im]­[TFSI], consistent with the stronger low-*q* composition fluctuations. Consistent with pure ILs, we also observe the formation of apolar domains with [C8C1im]­[TFSI], which corresponds to the prepeak at *q* = 0.3 Å^–1^. Overall, the MD snapshots help illustrate the structural interpretation of the three different SAXS features observed in these systems.

**Figure 8:**
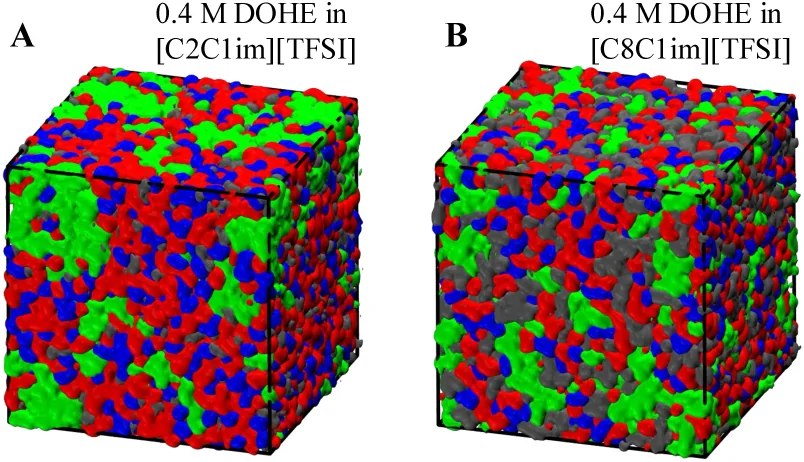
Snapshots of 0.4 M DOHE in [C2C1im]­[TFSI] (A) and [C8C1im]­[TFSI] (B). Blue: cation headgroup. Gray: alkyl chain on cation. Red: anion. Green: DOHE.

### 3.5 Pairwise *S_αβ_* (*q*) Contributions

Simulated SAXS provides an opportunity to decompose the scattering profile into contributions from interactions between individual components, thereby offering insight into how the overall liquid structure emerges from component specific organization. Having established that the simulated total SAXS profiles from MD qualitatively capture all features of the experimental measurements, we proceed to use the MD trajectories to decompose the scattering into pairwise structural contributions between different components of the IL and DOHE. We implement an established methodology for resolving the structure of IL mixtures by grouping atomic scattering contributions.^28,55^ Because both the IL and DOHE contain polar and apolar moieties, each component is divided accordingly. The imidazolium ring and TFSI anion are assigned to the IL polar region, while the cation alkyl tail is assigned to the IL apolar region; similarly, the DOHE headgroup and alkyl tails are assigned to the DOHE polar and apolar regions. The DOHE headgroup is defined to include the carbonyl groups, the α-carbon and the first carbon attached to the α-carbon, and the amine groups and the first carbon attached to each amine group. The remaining carbon atoms are assigned to the apolar tail group. The scattering contributions from these four groups are summed to calculate the partial structure factor, *S*_*αβ*_(*q*) (see eqs 3–5).

Structural features from bulk demxing and nanostructure manifest in different *q*-regimes and partial structure factors. First, we combine the polar/apolar partial structure factors for each molecule type to show the correlations between IL–IL, DOHE–DOHE, and DOHE–IL on a molecular basis, as plotted in Figure 9A–C. The dominant low-*q* feature of the partial structure factors is the segregation of DOHE and the IL, resulting in DOHE–DOHE and IL–IL correlation and DOHE–IL anticorrelation. This pattern is characteristic of composition fluctuations and indicates a tendency toward bulk demixing between DOHE and IL.

**Figure 9:**
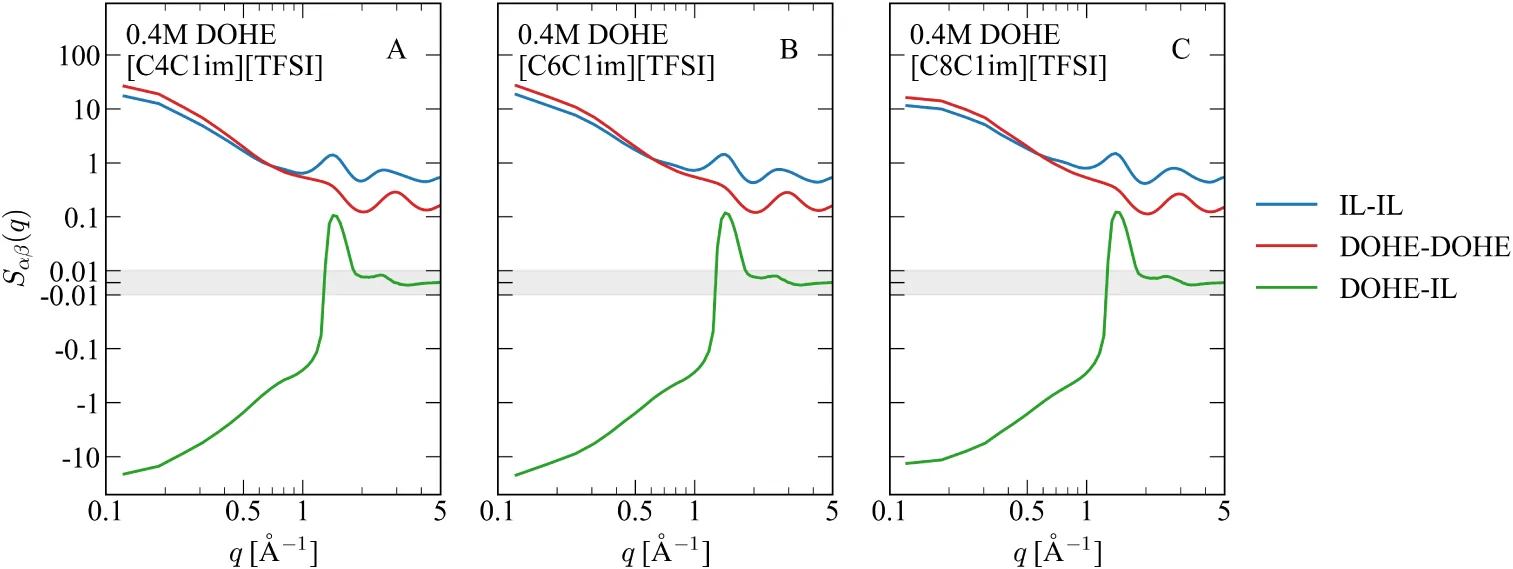
Pairwise structure factor contributions, *S_αβ_* (*q*), for 0.4 M DOHE in [C4C1im]­[TFSI] (A), [C6C1im]­[TFSI] (B), and [C8C1im]­[TFSI] (C). Panels A–C show the IL–IL, DOHE–DOHE, and DOHE–IL contributions by molecules. The *q*-axis is logarithmic, and the *S_αβ_* (*q*)-axis is symmetric logarithmic. The gray-shaded region, −0.01 ≤ *S_αβ_* (*q*) ≤ 0.01, denotes the linear transition region of the symmetric logarithmic scale.

While the demixing instability occurs between DOHE and IL as whole molecules, the incorporation of DOHE into the IL nanostructure is be better understood by considering the polar/apolar decompositions of DOHE and IL, plotted in Figure 10A–C. The cross term decomposition further tests this hypothesis by resolving the DOHE–IL interactions at different length scales. The apolar domain correlation (*q* ∼ 0.3 Å^–1^, see Figure 2) appears strongly in the DOHE apolar–IL apolar partial structure factor (red line), indicating that the DOHE tails associate with the IL cation alkyl tails that comprise the IL apolar domains. This correlation becomes sharper with increasing alkyl length of the imidazolium cation, while also shifting toward lower *q*, indicating larger and more defined IL/DOHE apolar domain sizes, consistent with behavior of the pure IL.^28^ At higher *q* corresponding to the charger alternation region (*q* ∼ 0.8 Å^–1^), the other DOHE and IL partial structure factors show weaker correlations suggesting some structuring of the DOHE with the charge domains. Despite being masked by the negative correlation associated with the demixing, these features reveal detectable correlations involving the polar and apolar regions of DOHE and the IL. The polar DOHE contribution shows shoulder-like features with both polar and apolar regions of the IL. Meanwhile the DOHE apolar-IL polar feature in this region mirrors the DOHE apolar-IL apolar feature with increasing correlation intensity as the IL domain becomes more well-defined for the longer IL cation tails. Lastly, the strong positive DOHE–IL peak near the adjacency region (*q* ∼ 1.3 Å^–1^) indicates nearest-neighbor interactions between DOHE and the IL. These features are consistent with the amphiphilic nature of DOHE: it can associate with charged regions of the IL, while its alkyl tails distribute into the apolar domains formed by the IL cation. Given that we only consider variation in the apolar tail of the IL cation (keeping same anion and same cation headgroup), our results most clearly highlight the impact of that tail length on controlling the strength of incipient bulk demixing through composition fluctuations and alignment of amphiphilic DOHE into the nanoscopic polar/apolar domain structure of the IL solvent.

**Figure 10:**
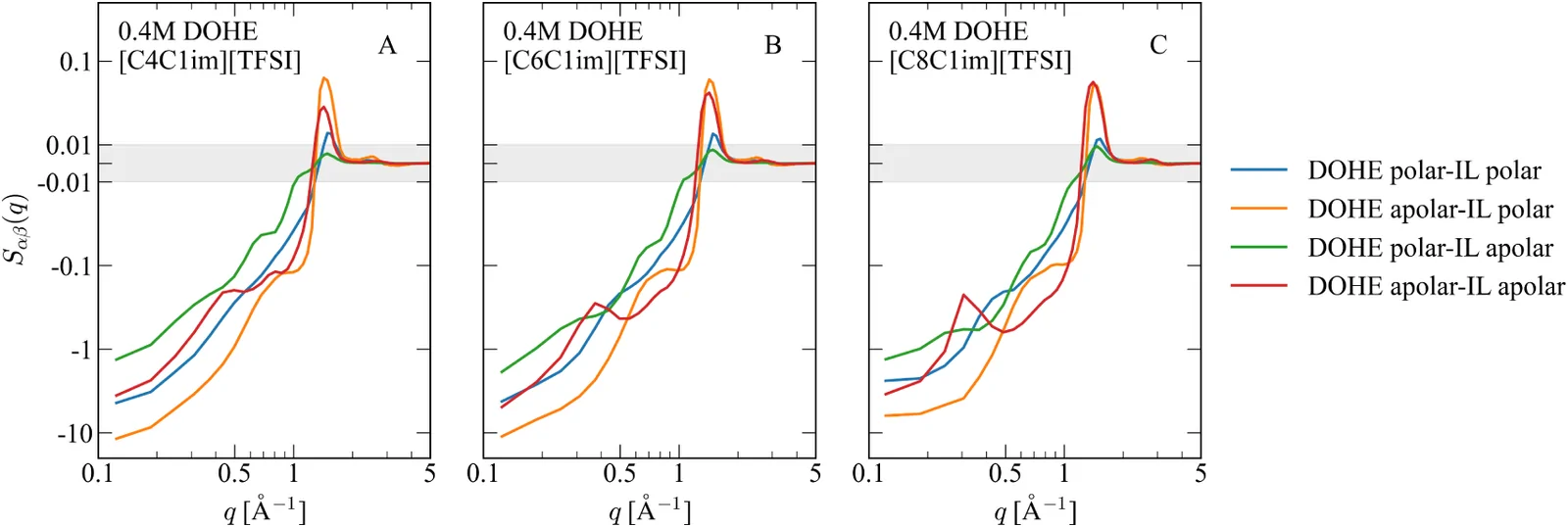
Pairwise structure factor contributions, *S_αβ_* (*q*), for 0.4 M DOHE in [C4C1im]­[TFSI] (A), [C6C1im]­[TFSI] (B), and [C8C1im]­[TFSI] (C). Panels A–C show the DOHE–IL cross contributions broken into the polar and apolar regions of DOHE and the IL. The *q*-axis is logarithmic, and the *S_αβ_* (*q*)-axis is symmetric logarithmic. The gray-shaded region, −0.01 ≤ *S_αβ_* (*q*) ≤ 0.01, denotes the linear transition region of the symmetric logarithmic scale.

## 4 Conclusion

In this work, synchrotron SAXS/WAXS measurements and MD simulations were combined to resolve the hierarchical liquid structure of DOHE/imidazolium IL mixtures across multiple length scales. The experimental and simulated scattering profiles show that adding DOHE introduces strong low-*q* composition fluctuations, especially in short-tailed ILs such as [C2C1im]­[TFSI], indicating proximity to liquid–liquid phase instability. OZ analysis shows that these fluctuations are concentration- and temperature-dependent, with the DOHE/IL mixture exhibiting UCST-like behavior with extrapolated spinodal temperatures below room temperature. Increasing the cation alkyl-chain length suppresses these fluctuations while enhancing the polar/apolar domain prepeak, demonstrating that longer IL alkyl tails better accommodate the amphiphilic DOHE molecules. Partial *S*_*αβ*_(*q*) decomposition from MD further shows that DOHE participates in both polar and apolar IL domain structures, while the strongest low-*q* feature arises from DOHE-IL composition fluctuations.

Our findings lead to a counterintuitive implication for ligand design strategies when using IL solvents. Most studies of LLE using hydrophobic ILs for the organic phase utilize conventional extractant molecules that were developed for typical (kerosene-like) organic solvents. A core trade-off in extractant design is the incorporation of nonpolar alkyl tails to improve the solubility of polar metal-extractant complexes in apolar media and thereby prevent polar/apolar phase demixing,^9,14^ while limiting the high viscosity of excessively long alkyl tails. This requirement typically favors increasing the size of the aliphatic tails of the extractant or decreasing the size of the aliphatic solvent. However, this trade-off is upended in the IL-based extraction systems. We instead find that the IL solvent is *more* polar than the extractant, such that smaller aliphatic portions of the solvent actually *decrease* the phase stability of the IL/extractant mixture. This implies that instead of needing to add carbon to extractant tails, when using ILs as solvents, one should remove carbon. The low-carbon limit would then likely result from the aqueous solubility of the extractant, which would negate its purpose of solubilizing ions in the nonpolar phase. It further suggests that the more polar extractant-metal complexes could be more stable in the IL than the less polar extractant itself. This could be related to the lack of third phase formation noted in IL extraction systems.^56^ Overall, these results suggest a complete reevaluation of the traditional trade-offs when designing extractant ligands for use with ILs.

## Supplementary Material



## Notes

Experimental scattering of different DOHE concentrations in [C3C1im]­[TFSI]; experimental scattering of different DOHE concentrations in [C4C1im]­[TFSI]; experimental scattering of 0.2 M DOHE in [C3C1im]­[TFSI] at different temperatures; experimental scattering of 0.2 M DOHE in [C4C1im]­[TFSI] at different temperatures; Comparison of Guinier and OZ fits to the low-*q* SAXS profiles of 0.4 M DOHE in [C2C1im]­[TFSI] and [C4C1im]­[TFSI] at 30 ^◦^C; residuals of fits for *ξ*_oz_(*T*) and *I*_oz_(*T*) using the 3D Ising and mean-field exponent sets; simulated *S*_*αβ*_(*q*), grouped by molecules, for 0.4 M DOHE in different ILs (PDF)

## #

Eichrom Technologies Inc., Lisle, IL 60532, United States
