# Supplementary material for: Unraveling Hierarchical Structure in Ionic Liquid–Extractant Mixtures with X‑ray Scattering
Source: J Phys Chem B. 2026 Oct 26;130(44):11866–74. doi: 10.1021/acs.jpcb.6c04769 (PMC13746780; doi:10.1021/acs.jpcb.6c04769)
Supplement: Supplementary file 1 [file jp6c04769_si_001.pdf]

Supporting Information: Unraveling Hierarchical Structure in Ionic Liquid–Extractant Mixtures with X-ray Scattering

Xiaoyu Wang,[*,†] Allison A. Peroutka,[‡,§] Soenke Seifert,[¶] and Michael J. Servis[*,‡]

†*Ralph E. Martin Department of Chemical Engineering, Univeristy of Arkansas, Fayetteville, AR 72701, United States*

‡*Chemical Sciences and Engineering Division, Argonne National Laboratory, Lemont, IL 60439, United States*

¶*X-ray Science Division, Argonne National Laboratory, Lemont, IL 60439, United States*

§*Current Address: Eichrom Technologies Inc., Lisle, IL 60532, United States*

E-mail: xiaoyuw@uark.edu; mservis@anl.gov

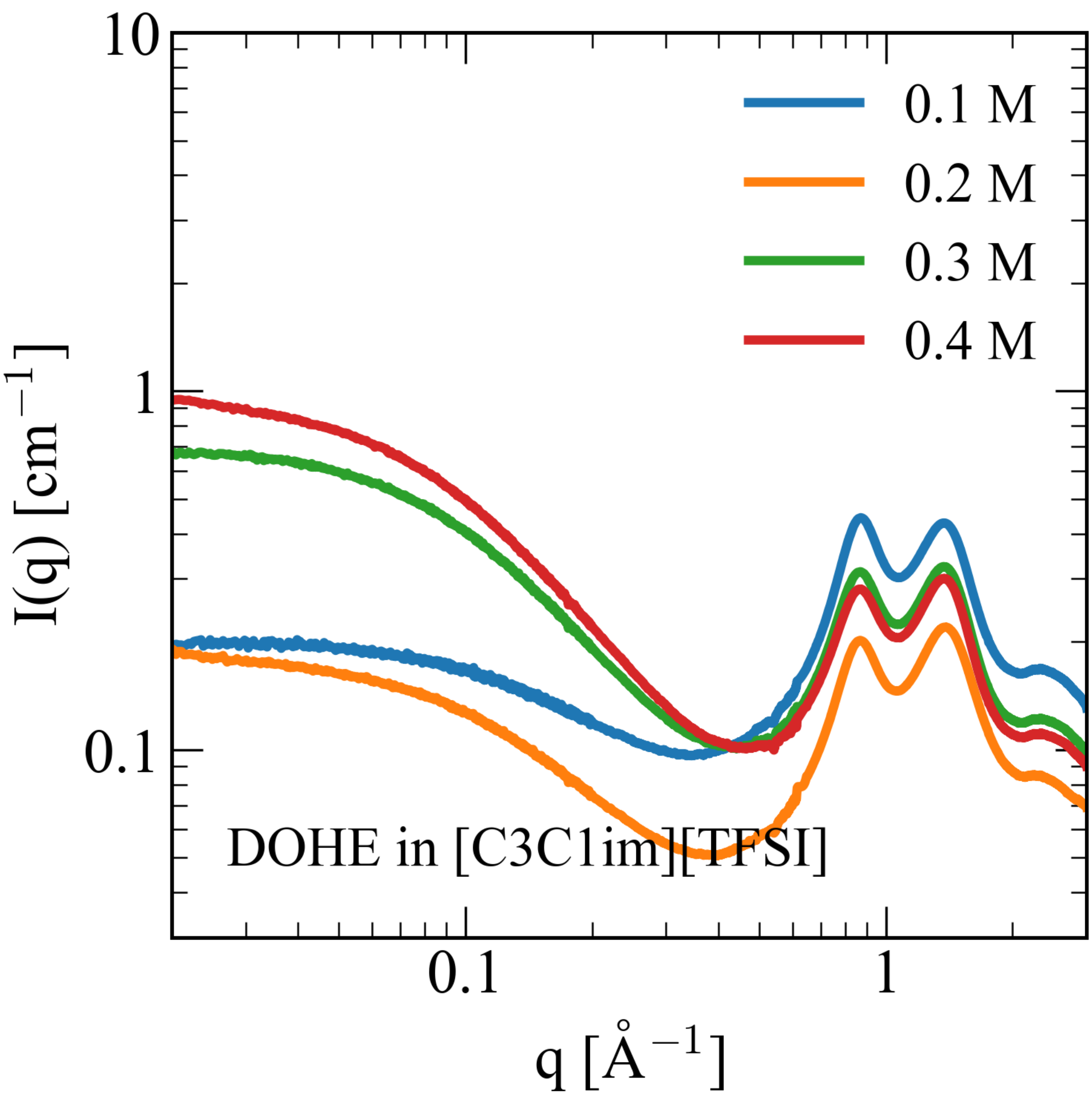

Figure S1: Experimental SAXS/WAXS scattering profiles of DOHE in [C3C1im][TFSI] at 25 °C for DOHE concentrations between 0.1 and 0.4 M.

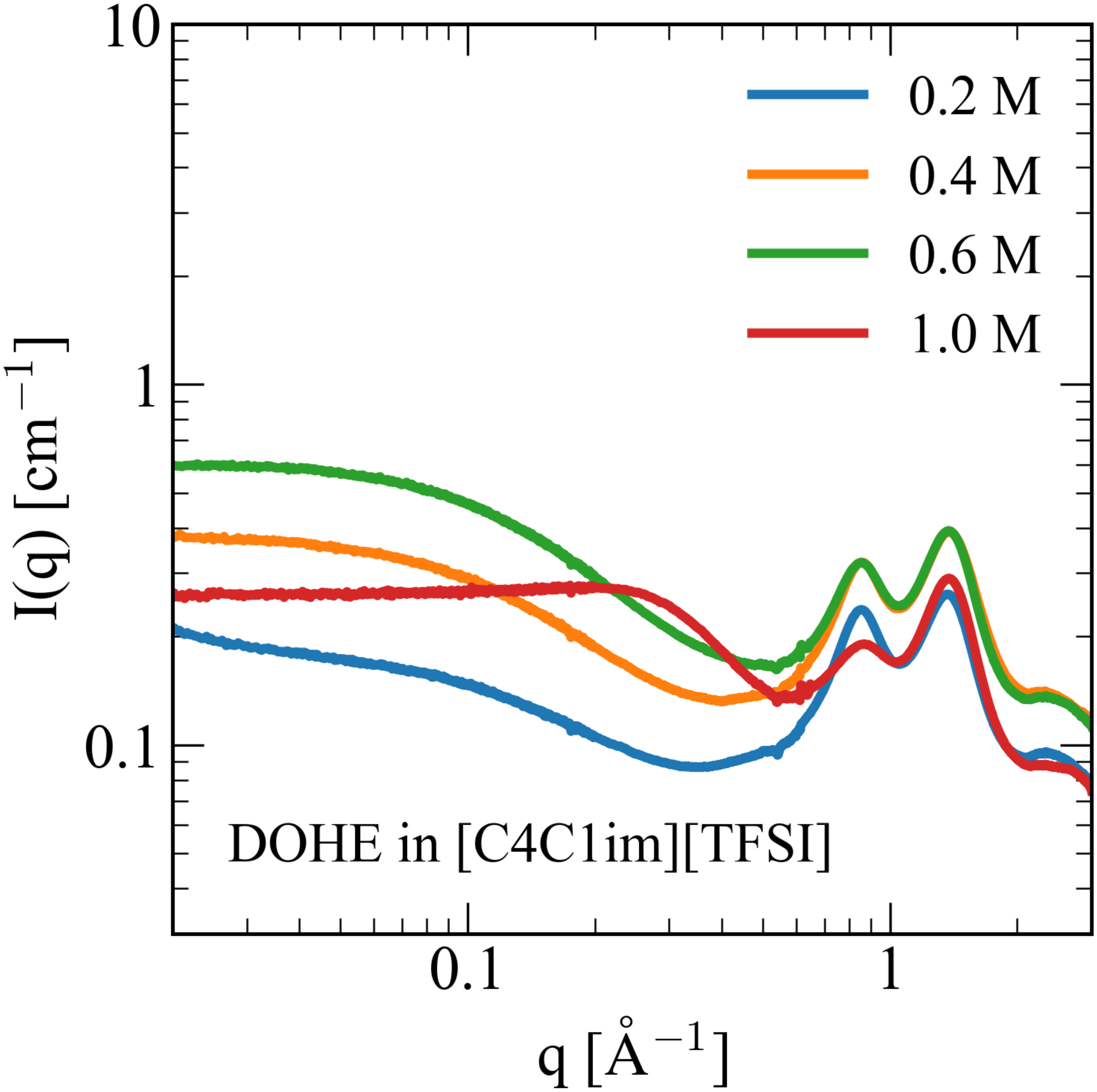

Figure S2: Experimental SAXS/WAXS scattering profiles of DOHE in [C4C1im][TFSI] at 25 °C for DOHE concentrations between 0.2 and 1.0 M.

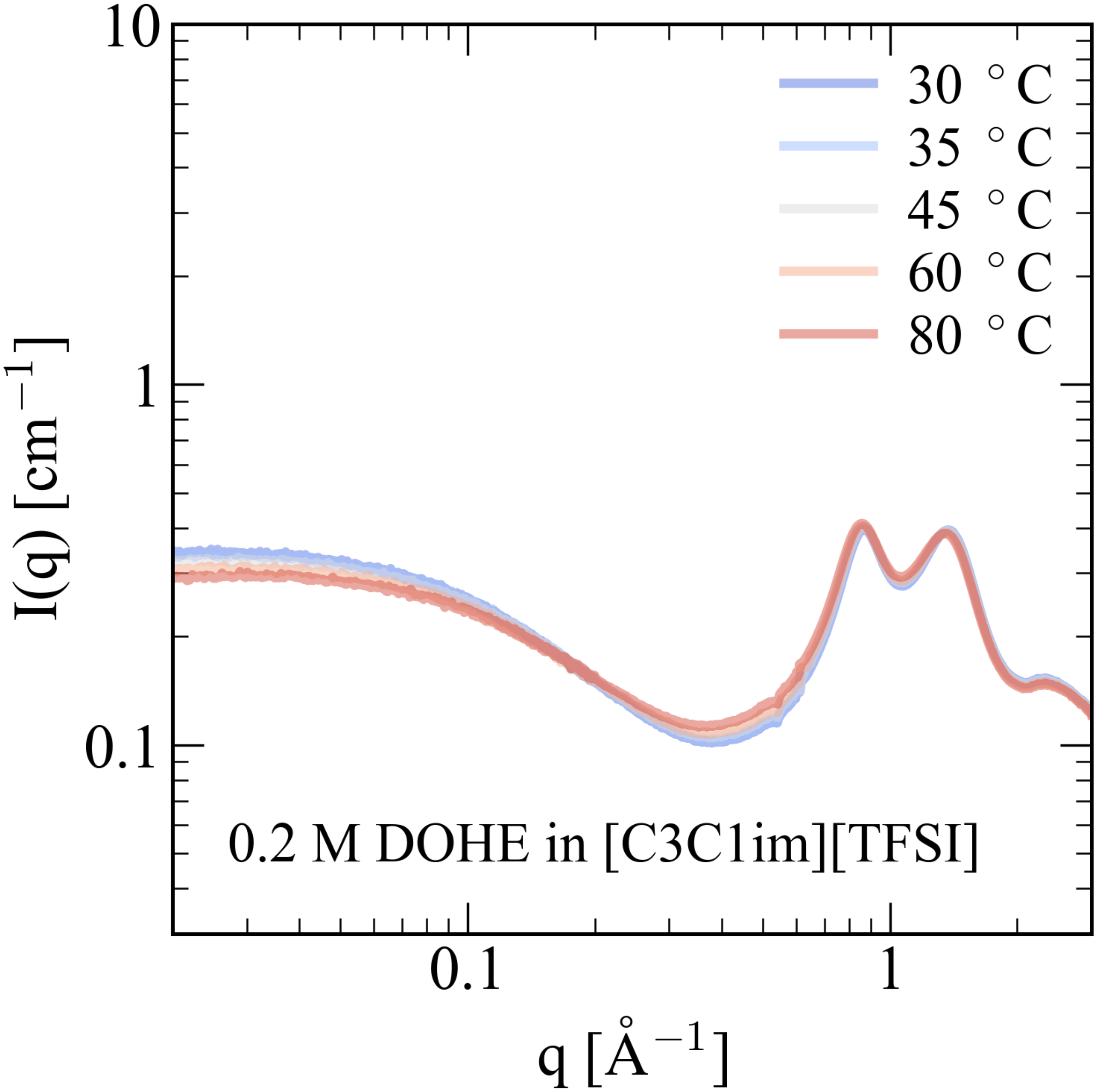

Figure S3: Experimental SAXS/WAXS scattering profiles for 0.2 M DOHE in [C3C1im][TFSI] measured between 30 and 80 °C.

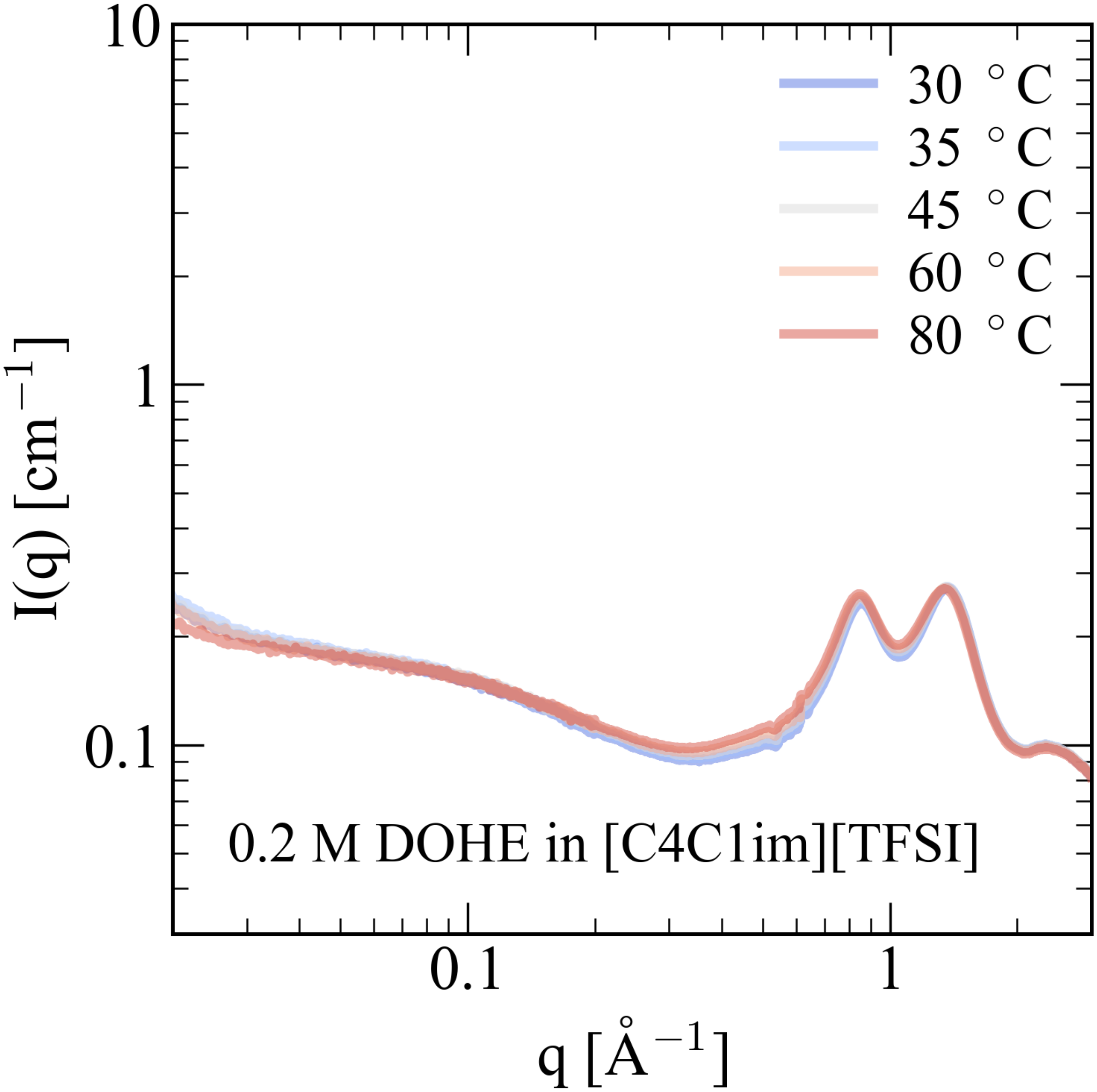

Figure S4: Experimental SAXS/WAXS scattering profiles for 0.2 M DOHE in [C4C1im][TFSI] measured between 30 and 80 °C.

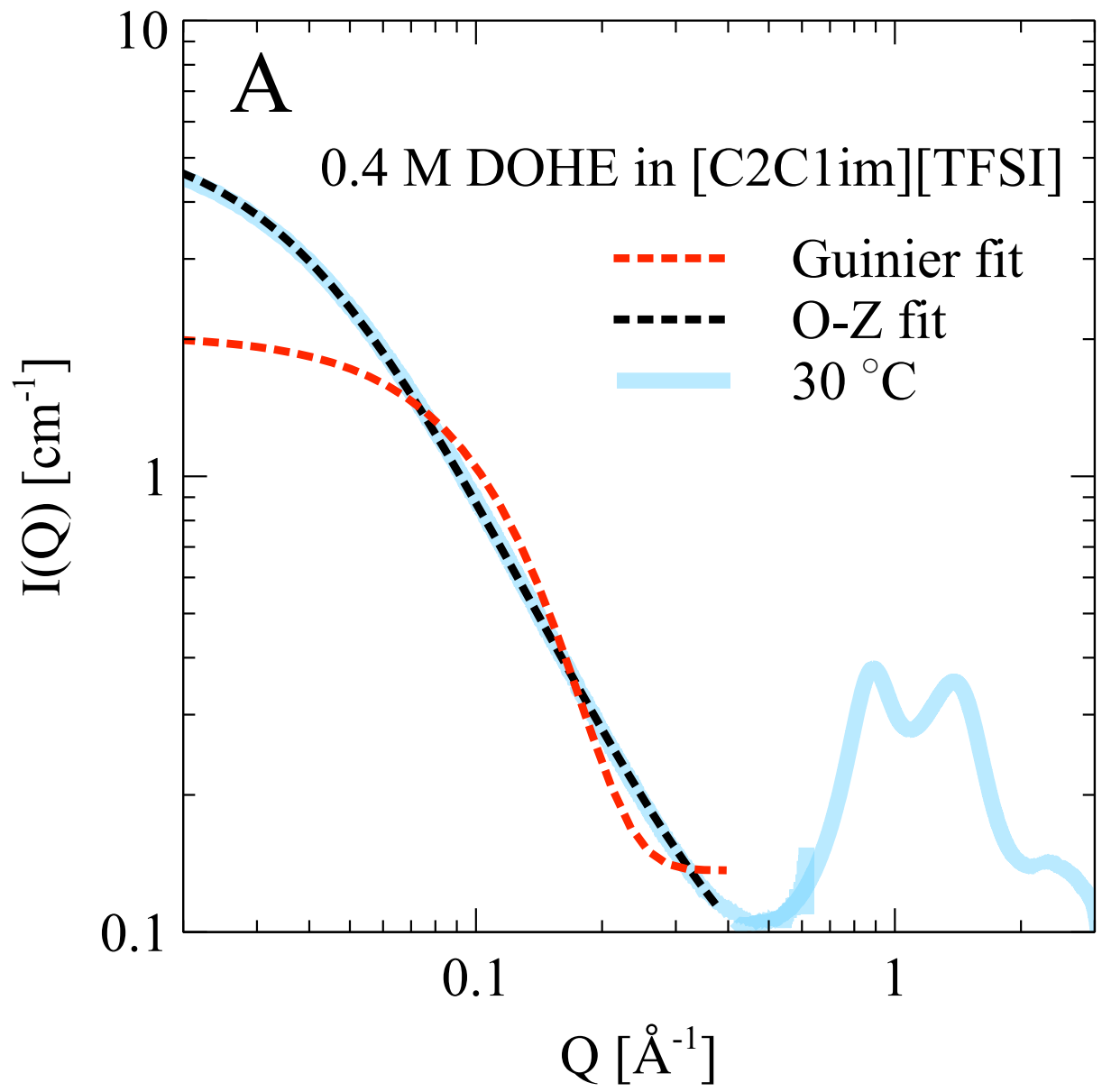

Figure S5: Comparison of Guinier and Ornstein-Zernike (O-Z) fits to the low-q SAXS profiles of 0.4 M DOHE in [C2C1im][TFSI] (left) and [C4C1im][TFSI] (right) at 30 °C. Experimental profiles are shown as blue solid lines, while the Guinier and O-Z fits are shown as red and black dashed lines, respectively.

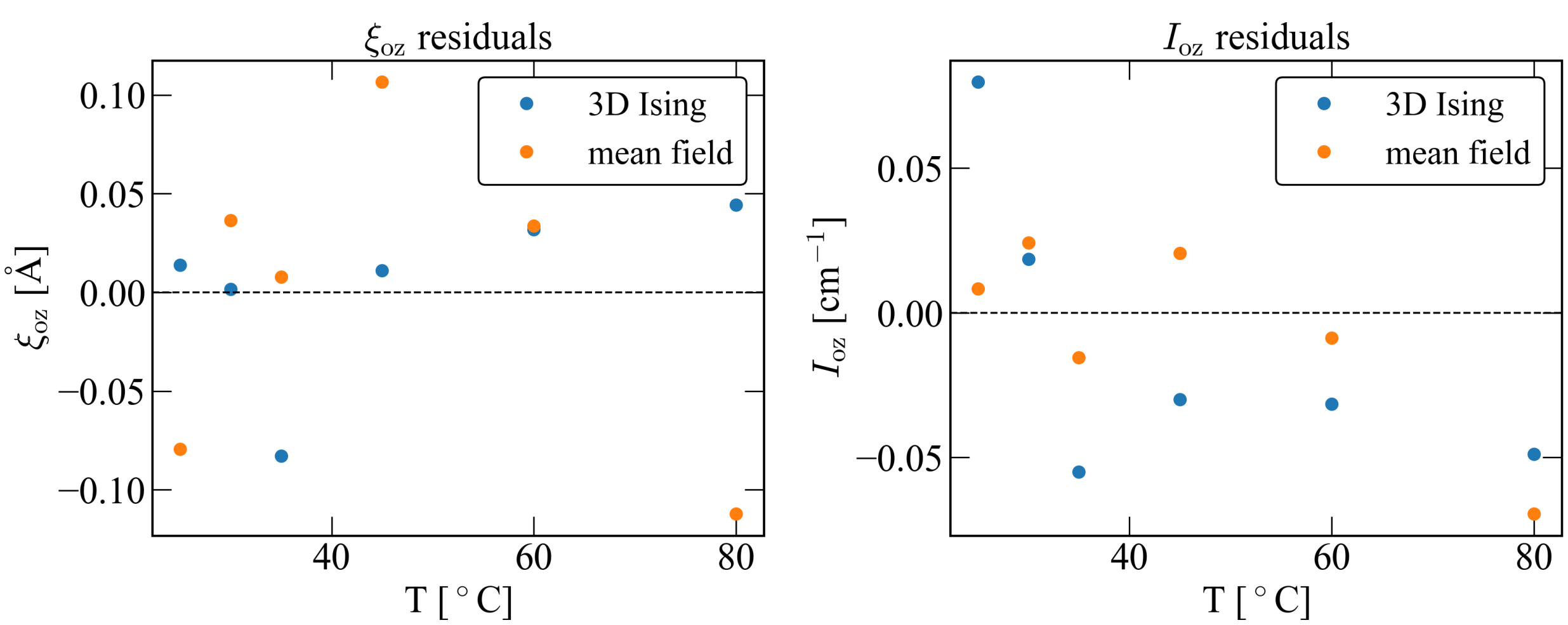

Figure S6: Residuals of the simultaneous scaling fits for ξ_{oz}(T) (left) and I_{oz}(T) (right) using the 3D Ising and mean-field exponent sets. Residuals are calculated as the measured value minus the fitted value.

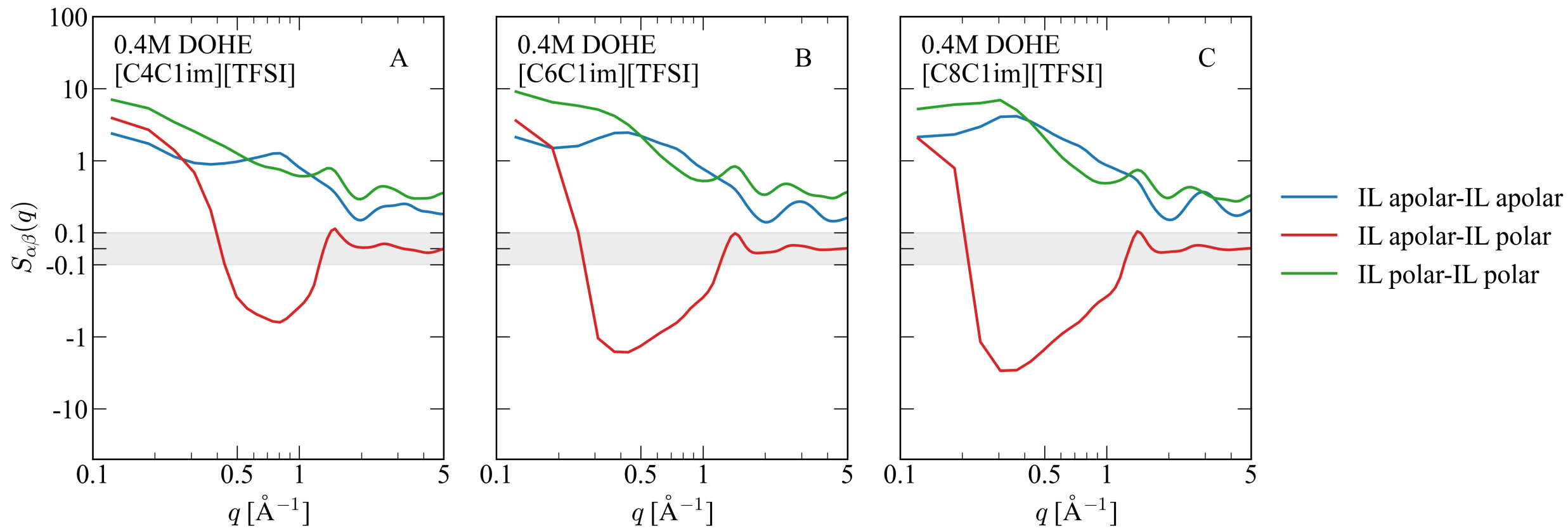

Figure S7: Pairwise structure-factor contributions, $S_{\alpha\beta}(q)$, for 0.4 M DOHE in [C4C1im][TFSI] (A), [C6C1im][TFSI] (B), and [C8C1im][TFSI] (C). Panels A–C show the IL-IL self contributions broken down into the polar and apolar regions. The q-axis is logarithmic, and the $S_{\alpha\beta}(q)$-axis is symmetric logarithmic. The gray-shaded region, $-0.1 \leq S_{\alpha\beta}(q) \leq 0.1$, denotes the linear transition region of the symmetric logarithmic scale.

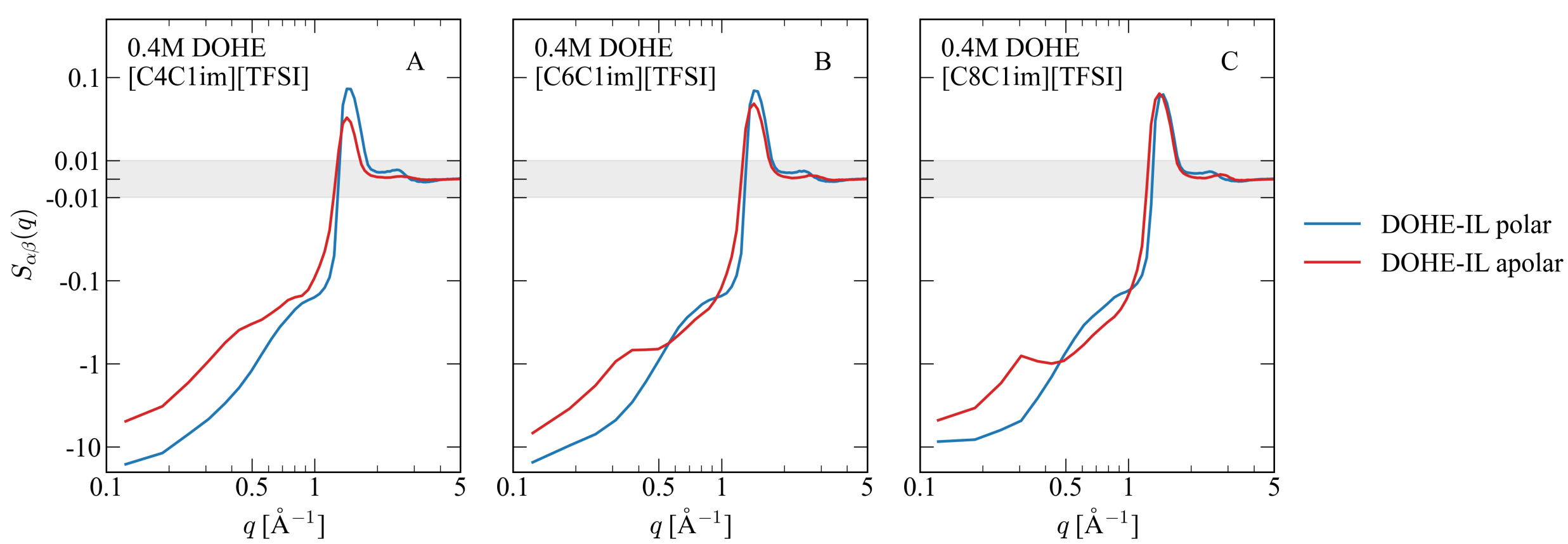

Figure S8: Pairwise structure-factor contributions, $S_{\alpha\beta}(q)$, for 0.4 M DOHE in [C4C1im][TFSI] (A), [C6C1im][TFSI] (B), and [C8C1im][TFSI] (C). Panels A–C show the DOHE–IL cross contribution between the whole DOHE and the polar and apolar regions of IL. The q-axis is logarithmic, and the $S_{\alpha\beta}(q)$-axis is symmetric logarithmic. The gray-shaded region, $-0.01 \leq S_{\alpha\beta}(q) \leq 0.01$, denotes the linear transition region of the symmetric logarithmic scale.
